# Quantitative Measurement of Vocal Fold Vibration in Male Radio Performers and Healthy Controls Using High-Speed Videoendoscopy

**DOI:** 10.1371/journal.pone.0101128

**Published:** 2014-06-27

**Authors:** Samantha Warhurst, Patricia McCabe, Rob Heard, Edwin Yiu, Gaowu Wang, Catherine Madill

**Affiliations:** 1 Faculty of Health Sciences, The University of Sydney, Lidcombe, NSW, Australia; 2 Division of Speech and Hearing Science, The University of Hong Kong, Hong Kong, China; Northwestern University, United States of America

## Abstract

**Purpose:**

Acoustic and perceptual studies show a number of differences between the voices of radio performers and controls. Despite this, the vocal fold kinematics underlying these differences are largely unknown. Using high-speed videoendoscopy, this study sought to determine whether the vocal vibration features of radio performers differed from those of non-performing controls.

**Method:**

Using high-speed videoendoscopy, recordings of a mid-phonatory/i/ in 16 male radio performers (aged 25–52 years) and 16 age-matched controls (aged 25–52 years) were collected. Videos were extracted and analysed semi-automatically using High-Speed Video Program, obtaining measures of fundamental frequency (*f0*), open quotient and speed quotient. Post-hoc analyses of sound pressure level (SPL) were also performed (n = 19). Pearson's correlations were calculated between SPL and both speed and open quotients.

**Results:**

Male radio performers had a significantly higher speed quotient than their matched controls (t = 3.308, p = 0.005). No significant differences were found for *f0* or open quotient. No significant correlation was found between either open or speed quotient with SPL.

**Discussion:**

A higher speed quotient in male radio performers suggests that their vocal fold vibration was characterised by a higher ratio of glottal opening to closing times than controls. This result may explain findings of better voice quality, higher equivalent sound level and greater spectral tilt seen in previous research. Open quotient was not significantly different between groups, indicating that the durations of complete vocal fold closure were not different between the radio performers and controls. Further validation of these results is required to determine the aetiology of the higher speed quotient result and its implications for voice training and clinical management in performers.

## Introduction

Radio performers are a group of professional voice performers for whom efficient vocal function and good vocal quality are integral to occupational success [1], [2]. This is reflected in a small body of literature which indicates that radio performers have better vocal quality, more variability in their fundamental frequency, a lower fundamental frequency and more low-frequency spectral gain than non-performing healthy controls [1]–[3]. The voices of effective speakers on radio are more likely to be characterised by a higher equivalent sound level (*L_eq_*) and lower smoothed cepstral peak prominence (CPPS) than the voices of radio performers and controls that are not reliably identifiable as good for radio [4]. These studies show that like other vocal performers, radio performers have different acoustic and perceptual characteristics from those of non-performing controls [1], [5]–[7].

Given that the acoustic and perceptual characteristics of the voice are manipulated at a physiological level – in vocal fold vibration or vocal tract shaping [8] – these studies imply that the vocal physiology of radio performers, actors and singers, differs from that of controls [6], [9]. To date, variations in the glottal source (e.g., vocal fold contact and glottal flow) in performers have been more easily and more frequently investigated than vocal tract manipulations, usually using electroglottography (EGG) or flow glottography [10]–[14]. Waveforms of glottal contact and flow collected using EGG and flow glottography respectively, have been analysed quantitatively using a range of different algorithms, yielding various results and interpretations [15]–[17]. Despite this variability, these studies have often utilised measures of vocal fold opening/closing rate (e.g., speed quotient (SQ),) and the durations over which the vocal folds are open and closed within each glottal cycle (e.g., open or closed quotients) [17], as they help determine the power and timbre of the sound [8]. These measures appear to reflect salient physiological features of voice production in all speakers, particularly vocal performers [15], [18], [19].

Although studies using waveforms of EGG and flow glottography have provided information about the vocal physiology of singers [16], our ability to generalise these findings to a broader category of vocal performers (inclusive of actors and radio performers) is limited by a number of factors. First, most studies have been conducted on small samples of singers, not spoken performers (e.g., [16], [20]). Moreover, although both EGG and flow glottography provide important information on glottal contact and airflow, they do not directly allow visualisation and measurement of vocal fold movement (e.g., as provided through laryngoscopy). This is shown in studies comparing waveforms from EGG, flow glottography and high-speed videoendoscopy (HSV; e.g., [17], [21]–[24]), which show weak relationships between the waveforms due to their differing modalities. This is probably because waveforms from EGG and flow glottography do not directly reflect a superior view of vocal fold kinematics, as do waveforms from HSV [17], [25]. In EGG, vibratory features are inferred based on electrical impedance measurements (reflecting vocal fold contact) [25] and inverse filtering of the oral airflow waveform in the case of flow glottography [17]. This means that same-named waveform measures (e.g., speed quotient) can differ significantly in what they measure across the different methods [17], [23]. For example, EGG displays only positive impedance when the vocal folds are making some contact (as depicted by Orlikoff and colleagues, [21]), so speed quotient measurements of an EGG signal reflect vocal fold contacting and de-contacting, during the closing and closed phase. This differs theoretically from HSV measurements of speed quotient, which reflect the ratio of vocal fold opening time to vocal fold closing time during the open phase [26].

High-speed videoendoscopy (HSV) allows direct and accurate visual capture of the intra-cycle vibratory behaviour of the vocal folds [27]. It has distinct advantages for visualisation and measurement of subtleties in vibration as well as of the irregular or aperiodic vibration usually associated with voice disorder [27], [28]. With frame rates up to 20 000+ frames/second commercially available, HSV has very high temporal resolution. This allows quantitative measurement of vibration symmetry, regularity, vocal fold edge, glottal closure, and mucosal wave as well as open and closed quotient [27]. The glottal area waveform (GAW) (often in pixels) has been used for quantitative examination of open quotient (OQ) and SQ as well as vibration symmetry, regularity and glottal closure in HSV images [29], [30]. A GAW is derived by delineating the glottal edge of each image frame [31] and turning these data into a plot of the glottal area over time [32]. Objective analyses of the GAW have been used in a number of studies of healthy voices and clinical cases [28], [31], [33], [34].

The High-Speed Video Program (HSVP) developed at the University of Hong Kong [28], [35] utilises measurements of the GAW to quantify HSV images. The HSVP has been used in examining the features of the GAW related to different types of voice production [28] and vocal fatigue [36] in healthy-voiced speakers. These results are consistent with other studies using qualitative and quantitative analysis of HSV, which found significant variability in ‘normophonic’ speakers, that is, those with no signs of dysphonia and/or with normal voice quality [21], [37]–[41]. Although those studies suggest that normal populations are heterogeneous for a range of physiological features, specific differences have not been reported to date between sub-groups of a normal population (e.g., vocal performers compared to controls, or voices with desirable perceptual features for a given performance context compared to voices without those features) for HSV-derived measures of vocal fold vibration.

Given that the physiological bases of many of the acoustic and perceptual characteristics of speaking voice performers have not been specifically isolated, this study sought to objectively compare features of the GAW as extracted from HSV-derived images in radio performers and non-performing controls. The study utilised the HSVP [35], as previous studies have shown that its quantitative measures are sensitive to variations in phonation mode and vocal function in normophonic speakers [28], [36]. Of particular interest are the program's temporal measures as taken from the GAW: *f0*, OQ and SQ. These measures provide important information about temporal aspects of the waveform from HSV images [26] and rely less heavily on spatial measurements of the GAW (compared to measures of glottal area and amplitude), which can be affected by uncontrolled magnification and endoscope position factors [36]. The HSVP uses well-established definitions of *f0*, OQ and SQ [29], [39], [42], and measurements appear to have high reliability [36].

This study used OQ, a measure of the relative durations of the open phase and the period of the glottal cycle [17], and SQ, the duration of the opening phase divided by the duration of the closing phase [17]. Both these measures originated from studies using HSV by Timcke and colleagues in the late 1950s [29], [42]. Since then, they have predominantly been applied to glottal waveforms derived from other voice assessment methods such as electroglottography and flow glottography [15], [17], phonovibrography [43], and kymography [44]. Previous studies using these temporal measures with electroglottograms and flow glottograms have shown that they may vary with changes in vocal timbre, pitch, loudness and physiology [8], [15], [17], [18], [45], [46]. However, the relationships between both OQ and SQ and discrete features of voice production are not always consistent. For example, a higher OQ suggests that the vocal folds are open for longer within each glottal cycle (i.e. a shorter closed phase duration). It provides a direct representation of the duration of glottal adduction [8] and generally changes with variation in both *f0* and intensity [26], [45], [47]. It is also higher in the falsetto register than in the modal register, in untrained male speakers [22]. Similarly, SQ (i.e., the duration of closing phases) often increases with increases in vocal intensity [26] but it also has a relationship with the increased vocal fold adductory forces seen in ‘pressed’ voicing [15], [47]. Recently, speed quotient values, when measured using kymography from HSV, were reported to vary significantly in healthy-voiced, male participants [19]. However, the impact of this variability on voice quality was not postulated. Therefore, although both OQ and SQ appear to be valid and reliable measures of vocal function and physiology [43], [45], interpretation of any OQ and SQ results needs to be made carefully and with respect to other vocal features such as fundamental frequency and intensity.

Thus SQ and OQ taken from HSV are potentially sensitive to differences in vocal fold kinematics between radio performers and controls. Speed quotient and OQ reflect glottal settings such as a shorter glottal closing time compared to opening time, and a longer glottic closure duration respectively. These settings have been suggested as potential contributors to acoustic features commonly seen in performers, such as a higher *L_eq_* and a more gradual spectral tilt [15], [18], [48], [49]. In the current study, therefore, the use of the HSVP to measure *f0*, OQ and SQ in radio performers allowed direct and accurate exploration of temporal aspects of vocal fold vibration in radio performers' voices. Previous studies have shown that radio performers have better (less dysphonic) voice quality than controls [2] and that good voices for radio have higher *L_eq_* than those not rated as good for radio, in a radio performance context [4]. It was hypothesised, therefore, that radio performers would have a higher SQ (which potentially contributes to a better vocal quality and possibly a higher *L_eq_*) and a lower OQ (reflecting a shorter open phase and longer closed phase and therefore a less breathy voice quality) than matched controls.

## Method

Ethical approval was obtained from the University of Sydney Human Research Ethics Committee (13089). All participants gave informed, written consent prior to their participation and this consent procedure was approved by the committee.

### Participants

Male radio performers (announcers, broadcasters, newsreaders and voice-over artists) aged 18–55 years with no history of voice disorder in the preceding year, at least one year's experience on radio, and with a self-reported Australian accent were recruited. Radio performer participants were recruited via email distributed by Commercial Radio Australia (a peak industry body), small and large commercial radio networks, voice casting agencies, public broadcast organisations and a radio advertising company. Twenty-three male radio performers aged 25–52 years volunteered for the study and met the inclusion criteria. As well, 31 male, non-performing controls aged within one year of the radio performer participants were recruited for the study through the University of Sydney's student and staff email bulletins and email advertisements sent directly to University of Sydney speech pathology students. Control participants were included if they had no history of professional (paid) vocal performance, no self-reported history of voice disorder in the preceding year, and an Australian accent. Female radio performers and controls were not included in the study due to limited recruitment of female radio performers and an insufficient sample size for statistical power.

### Data Collection Procedure

Recruited participants were invited to participate in an assessment of their vocal fold vibration using HSV. Three male radio performers did not consent to the procedure. The remaining participants underwent laryngoscopy using a 70° rigid endoscope attached to a Richard Wolf GmbH (Knittlingen, Germany) 5562 digital high-speed system. Videos were collected in colour at a frame rate of 4000 frames per second where 2 seconds of image capture was possible. Participants produced an /i/ sound with their tongue protruded, using a vocal pitch, loudness and quality that was as close to their habitual phonation as possible. Participants were not instructed to use their performance voice (i.e., as used for radio), as pilot participants found this task difficult with a rigid laryngoscope in place. Moreover, given that the phonation position was already unnatural, it was felt this would be counterproductive to obtaining a good image. When tolerated, two 2-second recordings of mid-phonatory /i/ (i.e. excluding onset and offset) were taken for each participant, and the video with the most complete view of the whole vocal fold length and the best image quality was chosen for further analysis. Participants who did not tolerate the laryngoscope well or for whom the full glottal length could not be viewed in at least one recording were excluded. Of the 20 male radio performers from whom laryngoscopy was attempted, 17 tolerated the procedure well enough for videos of adequate quality for further analysis to be obtained. Three radio performers were excluded as they did not tolerate the laryngoscopy procedure well and their videos were considered by the first and fifth authors (SW and GW) to be of inadequate view or length for further analysis. To control for age as a confounding variable, the radio performers were then matched with control participants of the same age (within +/− one year) for whom adequate videos were available. Age-matched controls for one radio performer could not be found in the sample of control participants with adequate videos. Therefore, videos for 16 male radio performers (mean age = 36 years, S.D. = 9 years, range = 25–52 years) and 16 age-matched controls (mean age = 35 years, S.D. = 9 years, range = 24–51 years) were included in further data preparation and analysis.

### Video Preparation and Analysis

A video preparation procedure was designed based on the procedure used by Yiu and colleagues [36] and is detailed below. All steps were initially performed by the first author (SW), and reliability for all steps is reported in the section that follows.

#### Extraction of frames for analysis and cropping of extracted videos

The videos were segmented to a length of 1000 frames (a segment length equal to or greater than previous research, e.g., [19], [30]) using Virtual Dub Portable Version 1.9.7 (Lee, 2005). For each participant, a segment with the least camera movement, the most constant light and the most consistent view of the vocal fold length was extracted and saved. No analysed segments contained vibration onset or offset.

The extracted videos were then cropped using the ‘null transform’ function in Virtual Dub Portable. The purpose of cropping was to make the original video files (256×256 pixels) more suitable for motion compensation, that is, by removing other moving structures that could confuse motion compensation of the glottis, such as the epiglottis, pyriform sinuses and aryepiglottic folds. Although this process reduced the resolution of the images from a resolution that was already relatively low, it did not change the resolution of the glottis, which was determined at the time of recording.

#### Motion compensation

For each video segment, an automatic motion compensation function built into the HSVP (run through MATLAB Version 2010b) was used to track and adjust the dynamic motion of the glottis due to endoscope movement. The motion compensation procedure also automatically converted each video to greyscale.

#### Video rotation and placement of analysis window

**Figure 1:**
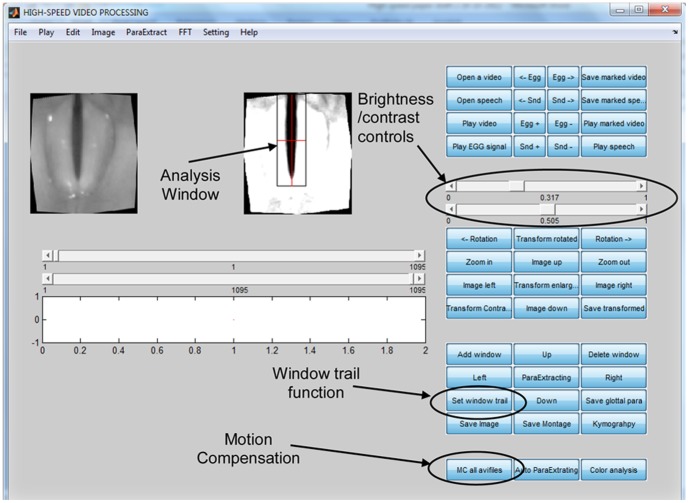
The High-Speed Video Program Graphical User Interface.

Using the HSVP, each video was rotated so that the longitudinal axis of the glottis was aligned with the vertical axis of the viewing window (as seen in Figure 1). Following rotation, an analysis window was dragged onto the maximally-opened glottis such that the edges of the window were in line with the left, right, anterior and posterior edges of the glottis. Even after the motion compensation procedure, some images appeared to have some slight residual endoscope motion, so the ‘window trail’ function was used to track any glottal motion. The ‘window trail’ function allows the user to place the analysis window into an appropriate location at the beginning and end of the video and the analysis window progressively moves between the two locations during the analysis.

#### Adjustment of brightness and contrast

Following placement of the analysis window and before extraction of the GAW, the brightness and contrast of each image were adjusted so that the glottal area was black and the vibrating vocal folds were white. To do this the operator moved the brightness and contrast controls, so that the black glottal area most accurately reflected the true glottal area in the unmodified image. Some videos required more brightness and contrast adjustment than others, depending on lighting levels and any reflections in the original HSV image.

#### Parameter extraction

The ‘auto parameter extracting’ function of the HSVP was used to perform the analysis. This function automatically converted the pixels within the analysis window to black and white, assisted by the brightness and contrast adjustments made in Step 6. The movements of glottal edges (white) were then automatically and continually tracked against the glottis (black) and measurements of the GAW were automatically calculated and exported to a Microsoft Excel spreadsheet.

### Parameters Used to Analyse the Glottal Area Waveform

**Figure 2:**
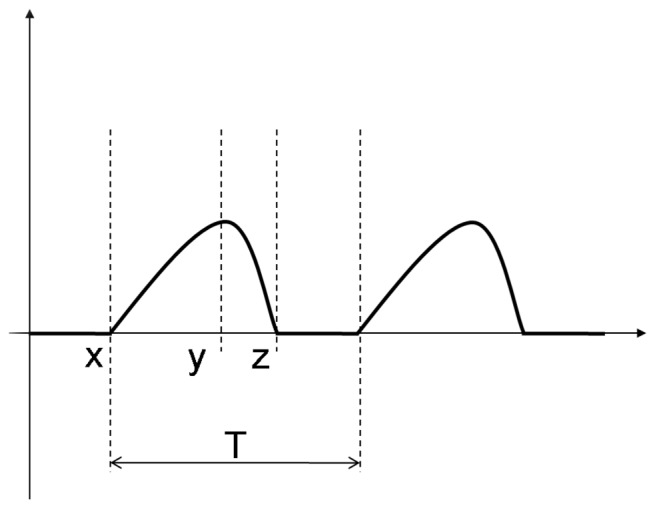
Diagram of Simulated GAW Showing Relevant Markers for *f*0 ( = 1/T), OQ ( = XZ/T), SQ ( = XY/YZ).

The HSVP can calculate a number of spatial and temporal measures. Currently, it is difficult to account accurately for magnification factors (i.e. exact zoom of camera, distance between vocal folds and laryngoscope) and accurate methods for extracting spatial parameters are still being determined. Therefore, three temporal measures of the GAW were calculated: *f0*, OQ and SQ. These were defined as follows [36] and are shown in Figure 2:

- *f0* – the inverse of the glottal period, measured from the GAW (1/T).
- OQ – the ratio of the duration of the glottal open phase to the duration of one full vibratory cycle. An average was taken for the total number of glottal cycles in the video. A higher average OQ suggests a relatively longer glottal open phase in each cycle.
- SQ – the temporal symmetry between the opening phase and the closing phase of the glottis during the open phase of the cycle, calculated by dividing the duration of opening by the duration of the closing within the open phase. The SQ was also calculated for each glottal cycle and the average SQ for all cycles in each video was used in statistical analysis.

It should be noted that HSVP automatically produces the OQ and SQ as percentages. However, for all participants these were converted to decimals (i.e., all values were divided by 100), for ease of comparison with other studies on OQ and SQ.

### Inter and Intra-rater Reliability

The video preparation phase above relied significantly on the subjective perceptual judgments of the HSVP user, which might have had an effect on the glottal waveform measures. Therefore, both intra- and inter-rater reliability were computed using the results of 12 videos (approximately 30% of the sample) selected at random, using the HSVP measures and the contrast/brightness settings as dependent variables. For intra-rater reliability, the first author (SW) re-analysed 12 random videos using the HSVP (i.e. repeating all data preparation steps) approximately 5 days after the initial analysis was performed. Similarly, for inter-rater reliability, the fifth author (GW) performed all steps of the video preparation and analysis procedure that utilised the HSVP (i.e., from video rotation onwards) on another 12 random video samples. For both intra- and inter-rater reliability, intra-class correlation coefficients (ICCs) were used to determine the agreement between the initial and re-analysed values for each dependent variable.

### Audio Recording and Sound Pressure Level Analysis

Previous research using SQ in both HSV and other glottal waveforms indicates that SQ may vary with changes in speaking intensity or equivalent sound level (*L_eq_*). That is, a higher SQ is usually associated with a higher *L_eq_* [45]. To determine any effects of a varying *L_eq_* on the hypothesised SQ and OQ results, we calculated *L_eq_* using audio recordings simultaneously collected during the endoscopy process. To do this, a Wolf 5052.801 microphone was mounted on the rigid endoscope and collected audio recordings during all video recordings. This created a mouth-to-microphone distance of approximately 10 cm. The Wolf system saved these recordings automatically with each video file for each of the 32 participants. Calibration of the sound levels of the recordings was also performed at this point using a sine wave tone generated in Soundswell version 4.5 [50] and a sound pressure level meter (TECPEL 331 Sound Level Meter). Given the capabilities of the Wolf system, however, it was not possible to view the audio recording during data collection. Following the laryngoscopy procedure, each audio file was inspected using *Wavepad* (NCH software, version 5.15) and it was found that recordings for 13 participants (7 radio performers and 6 control participants) had significant periods of clipping and were unsuitable for acoustic analysis. For the remaining participants (n = 19), a sustained portion of each vowel was extracted with a minimum length of 400 ms and maximum length of 1 s. These vowels were saved as wav files at 16 bits. It should also be noted that this analysis was done post-hoc (i.e., after the video analysis was performed). The audio samples analysed were from the same video segment analysed in the main study but the audio segments extracted could not be exactly synchronised with the already extracted videos. In light of this, a stable sustained vowel portion (i.e., greater than 300 ms from onset or offset if applicable) was selected for each participant.

The extracted wav files were then analysed for *L_eq_* using the Soundswell Histogram tool (version 4.5, [50]). Sound pressure level calibration was maintained and calibrated SPL values for each participant's audio sample (total n = 19) were recorded.

### Statistical Analysis

Differences between the radio performers and controls for *f0*, OQ and SQ were examined using independent samples t-tests (SPSS Version 21.0). Z scores for skewness and kurtosis were calculated to test for normal distribution in each dependent variable. A Bonferroni adjustment was performed to minimise Type I error, as three relatively new measures of HSV with unknown difference limens were tested. Therefore, a p-value of less than 0.016 (0.05/3) was considered statistically significant.

Given the previously documented relationships between OQ and SQ with sound pressure level (SPL), two Pearson's correlations between OQ and SPL as well as SQ and SPL were performed using both participant groups. Independent samples t-tests were used to examine differences in mean SPL between the radio performer participants and controls (for which SPL values were available; radio performers n = 9, controls n = 10). Bonferroni adjustments were not applied to these analyses.

## Results

### Reliability Analysis

Intra- and inter-rater reliability results for each dependent variable as well as the contrast-brightness settings are shown in Table 1. Both intra- and inter-rater ICCs showed good to excellent reliability (intra-rater ICCs = 0.869–1.000, inter-rater ICCs = 0.898–1.000).

**Table 1:** Intra- and Inter-Rater Reliability Results (Intra-Class Correlation Coefficients) for Manual Components of the HSVP.

Measure	Intra-Rater Reliability	Inter-Rater Reliability
*f0*	1.000	1.000
OQ	0.898	0.971
SQ	0.920	0.869
Brightness	0.926	0.938
Contrast	0.951	0.969

### Differences between Radio Performers and Controls for *f0*, OQ and SQ

Descriptive statistics for *f0*, OQ and SQ for the radio performers and controls are shown in Table 2. None of the variables departed significantly from normal skewness and kurtosis (Z scores between −2.575 and +2.575, p>0.01). The male radio performers had a significantly higher SQ than the controls (t = 2.795, p = 0.008). Cohen's d for this effect was approximately 0.93 (large effect), based on the pooled standard deviation. Differences between radio performers and controls were not significant for *f0* (t = −0.548, p = 0.587) and OQ (t = 0.649, p = 0.112).

**Table 2:** Descriptive Statistics for f0, OQ and SQ for Male Radio Performers and Their Respective Matched-control Groups.

Measure	Fundamental Frequency	Open Quotient	Speed Quotient*
Radio Performers	171 Hz (56 Hz)	0.68 (0.19)	1.08 (0.13)
Controls	194 Hz (65 Hz)	0.79 (0.17)	0.97 (0.09)

N.B. Means are reported, followed by standard deviations in parentheses. Although the HSVP provides open and speed quotient results as a percentage, results are presented here as a decimal for ease of comparison with previous literature. As a guide, an open quotient of 1 indicates no vocal fold closure and a speed quotient greater than 1 indicates a longer glottal opening compared to closing duration.

* =  significant measure, differentiating radio performers from controls.

### Correlation of OQ and SQ Variables with SPL Values

No significant correlations were found between OQ and SPL (r = −0.246, n = 19, p = 0.326) or SQ and SPL (r = 0.415, n = 19, p = 0.087).

### Difference between Radio Performers and Controls for SPL

No significant difference in SPL between the radio performer and control groups was found (t = 1.234, p = 0.235).

## Discussion

This study used HSV to investigate the physiological differences in vocal fold vibration between male radio performers and controls. The male radio performers had a higher SQ than the non-performing controls. Descriptive statistics indicated that the male controls had almost equal glottal opening and closing times, similar to normophonic subjects in previous research using videokymography [51]. In contrast, many of the male performers had a relatively longer opening phase and a shorter closing phase. These large differences in SQ, equating to a Cohen's d of approximately 0.93, occurred in the absence of any significant differences in *f0* or OQ. Furthermore, the correlation between SPL and SQ was weak and did not reach significance, suggesting that the difference in SQ between the participant groups was unlikely to be related to a difference in speaking volume.

Objective measurements of HSV-derived glottal waveforms are still in their infancy and significant discussion continues surrounding the interpretation and physiological significance of many measures [30]. Although *f0*, OQ and SQ are conceptually similar to their same-named counterparts in flow glottography [8], [46], EGG [17], phonovibrography [43] and kymography [51], they are not directly comparable to these other waveforms for methodological reasons (e.g., [21]). In previous studies using other glottal waveform measures (mostly EGG and glottal airflow waveforms), the physiological bases of f0, SQ and OQ have been discussed in detail but are used with caution in the interpretation of the current results.

A higher OQ shows that the vocal folds are open for relatively longer within each glottal cycle, and therefore have a shorter closed phase duration. Changes in OQ are influenced by the degree of adduction of the vocal processes (arytenoid cartilages), such that the OQ increases with abduction [8]. Perceptually, a higher OQ is generally related to breathiness (except in the case of ‘flow phonation’ where slight abduction of the vocal folds is desirable [20]) and a lower OQ is associated with a ‘pressed’ voice quality [46]. A higher OQ has been associated with a higher f0 in modal registers as well as with differences in vibration between modal and falsetto registers [26]. It also has a direct relationship with vocal intensity [45], [47]. However, these relationships are not consistently supported in the literature and are not consistent across waveform methodologies (e.g., in [24], [37]). For example, in the current study, there was no evidence of a relationship between OQ and SPL in the correlations performed for 19 participants.

In the current study, moreover, OQ values for radio performers and controls did not differ, indicating that the radio performers did not have a shorter open phase (or longer closed phase) than the controls. Although the possibility of a type II error cannot be excluded for this OQ result, the large effect for SQ indicates that, even if the OQ means differ between the two groups, the difference is considerably smaller than the significant SQ difference. Therefore, this OQ result is in contrast with previous acoustic and perceptual studies of speaking voice performers, which implied a longer period of vocal fold closure in the performer group based on a tendency for performers to speak at greater intensities [4], [26] and have better (including less breathy) voice quality [1], [2] than controls.

A higher SQ and an associated shorter closing phase seem to be physiologically related to the increased vocal fold adductory forces seen in pressed voicing [15], [47] as well as an increased subglottic pressure and vocal intensity [45]. Given the higher SQ result seen in radio performers in this study, it is possible that through inherited vocal features, training, experience or otherwise, their vocal folds closed more quickly or with greater recoil force than those of controls [15], [52], [53].

However, any further interpretation of the SQ seen in this study needs to be performed with the participants' SPL results in mind. This is because the relationships of both SPL and subglottic pressure with SQ are well documented, mostly for measurements of SQ taken from flow glottograms [17], [24], [45]. Post hoc analysis in the current study found no evidence of a relationship between SQ and SPL. However, the lack of correlational evidence may have been due to the reduced sample size used in the Pearson Correlation (n = 19) and the difficulty in exactly synchronising the audio and video segments analysed, so the conclusion must remain tentative. The non-significant SQ and SPL correlation found in this study could also be explained by the use of a HSV waveform in calculating SQ. Although a handful of studies have supported a relationship between HSV-derived SQ and SQ [29], [42], most studies of both SQ and SPL have used EGG or flow glottography in their investigations. As discussed in the introduction, SQ measures different behaviours across waveform methods and so it is possible that HSV-derived SQ, a measure of only the superior view of glottal vibration, does not change as directly with SPL as its EGG and flow-glottography-derived counterparts.

In developing this study, it was hypothesised that a higher SQ in radio performer participants might reflect one of the mechanisms that result in a higher *L_eq_* (SPL) in a performance context. A previous study on the same participants found that a higher *L_eq_* seemed to be a significant, perceptually desirable feature of radio performers' voices in the context of effective communication on radio [4]. Our results, however, found no evidence for this hypothesis; no relationship between SPL and SQ was found and no differences in SPL were found between radio performers and controls for the audio samples recorded during HSV collection. These observed differences in *L_eq_* across the two studies were likely due to differences in task, that is, the current study tested sustained vowels and the previous study tested performance-like connected speech. Further research is required to examine the vocal fold kinematics and other physiological manipulations used by radio performers in producing a higher *L_eq_*, particularly in a performance-like context.

Although the relationship between SQ and SPL is not supported by these results, a number of other acoustic and perceptual features may be related to a higher SQ. Firstly, the higher SQ result in radio performers reflects a skewed GAW, a phenomenon that has been associated with a more ‘brassy’ (less ‘fluty’) voice quality in previous research into glottal airflow waveforms [8]. Secondly, researchers have suggested both statistical and theoretical links between faster glottal closing speeds (which may result in a higher SQ value) and a more gradual spectral tilt [15], [54]. Thus the higher SQ in radio performers might be related to the production of a voice signal with more energy in the higher overtones and a more gradual spectral tilt. A gradual spectral tilt has been shown to be associated with good voice quality in actors [6], [49] but its salience for good voice quality on radio requires further investigation.

Further interpretations of this result are provided with caution, as the specificity of SQ in isolation is low and because most previous physiological interpretations of SQ results have been based on other glottal waveforms that did not involve direct visualisation. Given the previously-documented relationships between SQ, OQ, subglottic pressure, intensity and frequency [8], [10], [17], [45], further exploration of these results is required in a more controlled environment. For example, examination of radio performers' vocal fold kinematics using high-speed laryngoscopy, with measurements of both subglottic pressure and sound pressure level, would allow more specific interpretations to be made. However, these results suggest that voice production in radio performers may have be associated with a characteristic vocal fold vibration pattern, not seen in a non-performing population, which potentially increases their communicative effectiveness or perceptual desirability on air.

### Implications

These findings provide further confirmation of the role of the vibration ‘source’ (i.e. glottal settings such as glottal opening and closing durations) in the voice production of performers such as radio performers, actors and singers (as previously proposed in [15], [48], [49]). The higher SQ in our male radio performers suggests that the vocal fold vibration of performers is likely to be different (and possibly more optimal) than that of healthy, non-performing controls. Given the documented links between glottal closing speed (as reflected in SQ and other measures) and voice quality [8], [48], the results seen in this study may reflect the radio performers' need to sound warm, clear and animated [55]. However, the difference limen for speed quotient, as measured using HSV, has not been documented and so the perceptual salience of this difference in the context of radio communication requires further investigation.

Further research that examines the effects of specific clinical/voice-training cues on the glottal waveform (including vocal fold adductory patterns) would contribute significantly to our understanding of vocal function in performers. Moreover, examination of relationships between objective HSV-derived data and data referring to participants' previous training and background (e.g., from surveys) could provide further clues as to the aetiology of the higher SQ found. In the meantime, these results suggest that radio performers' voices are characterised by specific physiological features, namely a higher ratio of glottal opening to closing time. This should be considered in vocal training and clinical management of voice disorders, as this physiological modification has been shown to be salient to voice production in radio performers. For example, it may be useful to determine which training techniques explicitly facilitate efficient and healthy use of a higher SQ (e.g., through instruction in manipulating vocal fold mass, tension or subglottic pressure).

### Limitations

This study was conducted on a sample size of 32 male radio performers and controls, all of whom were from one geographical background (greater metropolitan Sydney). The sample size was of particular issue when investigating the relationship between the HSV variables and SPL (n = 19 for this comparison), so more definitive research on correlations between these measures should be conducted with a larger sample size. Further, research with other English and non-English speaking populations could determine whether this phenomenon is seen in radio performers from different geographical and linguistic backgrounds. Research with female participants is also required, to provide information on whether the higher SQ finding from this study also applies to performers with a different vocal mechanism (i.e. a smaller vocal fold mass).

This study used only three temporal measures that examined gross differences between the radio performers and controls. These measures came from a relatively new program, the HSVP, and require further validation with a larger dataset and against measures of HSV waveforms from other programs. Further, other measures of perturbation (e.g., as used by Kunduk and colleagues and Inwald and colleagues [43]) as well as spatial measures may provide more sensitive measurement of vocal fold vibration in vocal performers. Finally, although HSV is a powerful tool for capturing features of a sustained vowel, rigid laryngoscopy is invasive and the phonation position is highly unnatural, for which reasons functional information relevant to vocal performance might be lost in such a task. The degree of unnaturalness involved in the task can be seen in the *f0* results for both groups, which are higher than the normal range for males in sustained vowels. It is recommended that further study of vocal fold vibration in performers be performed using high-speed flexible nasendoscopy, similar to the setup used by Echternach and colleagues [56], to facilitate a more natural voicing condition. This would also allow concurrent collection of subglottic pressure data and a more natural audio signal, if required, and would address limitations of the current studies. More rigorous investigation of the relationship between objective features of vocal fold vibration and perceptual attributes of voice would also be possible using a flexible HSV setup.

## Conclusions

This study used HSV to examine temporal aspects of vocal fold vibration in both male radio performers and healthy, non-performing controls. The male radio performers were found to have a higher SQ than the controls, suggesting different vocal fold kinematics in the two groups, which might be linked to their occupational requirements. Further exploration is warranted of the relationships between physiological adjustments of the vocal folds (including glottal closing duration), voice-training cues and good voice quality in radio and other voice performance activities.
